# Evaluating the Cost-Effectiveness of Comprehensive Geriatric Assessment: Protocol for a Systematic Review of Economic Evaluations

**DOI:** 10.12688/hrbopenres.14066.2

**Published:** 2025-11-11

**Authors:** Amanuel Yigezu, Rose Galvin, Dominic Trépel

**Affiliations:** 1 Trinity College Institute for Neuroscience, Trinity College Dublin, Dublin, Ireland; 2 School of Medicine, Trinity College Dublin, Dublin, Ireland; 3 School of Allied Health, Ageing Research Centre, Health Research Institute, University of Limerick, Limerick, Ireland

**Keywords:** Comprehensive geriatric assessment, Economic evaluation, systematic review

## Abstract

**Background:**

Comprehensive Geriatric Assessment (CGA) is a multidimensional interdisciplinary diagnostic process focused on determining an older person's medical, psychological, and functional capabilities to inform a coordinated and integrated health plan. It is a multifactorial intervention requiring longitudinal planning and care coordination, which can utilize variable healthcare resources and have difference health outcomes. This systematic review aims to identify evidence of the cost-effectiveness of CGA in various care settings.

**Methods and analysis:**

Full economic evaluation studies (comparing both the costs and the consequences of alternatives) on CGA will be searched for in the Embase, Medline, CINAHL, CEA registry, and NHSEED databases. Two independent reviewers will screen the studies against the eligibility criteria and extract data using a pretested extraction form. We will include either randomized control trials or model-based economic evaluations, and the outcomes will include the mean costs and effectiveness, incremental cost, and incremental effectiveness. Reporting quality will be assessed using the Consolidated Health Economic Evaluation Reporting Standards (CHEERS-2022) checklist. Narrative summary tables and figures will be used to present the study characteristics.

**Ethics and dissemination:**

Ethics approval is not required for this systematic review because we will only utilize publicly available economic evaluation studies rather than individual patient data. The findings of this review will be presented at national and international conferences and published in peer-reviewed journals.

PROSPERO registration no.: CRD42023492586.

## Background

With increasing life expectancy and changes in demography ^1^, the demand for healthcare is increasingly rapidly in line with the growth of the older population ^2^. The *World Health Organization (WHO)* defines "healthy ageing" as " *the process of developing and maintaining the functional ability that enables well-being in older age*" ^3^. To realize healthy aging, health and social care systems advocate a shift from acute management of cases coordinated, longitudinal, and integrated care for older adults ^4^. As a result, researchers are increasingly focused on identifying interventions that improve healthy ageing.

Comprehensive Geriatric Assessment (CGA) is a multidimensional interdisciplinary diagnostic process focused on determining an older person's medical, psychological, and functional capabilities to develop a coordinated and integrated care plan ^5^. CGA is considered for older adults living with frailty or at risk of adverse outcomes ^6^. To improve the trajectory of aging, CGA considers various factors, including medical, psychological, and functional impairment, as well as environmental and social issues. CGA requires an interdisciplinary team that may include geriatric specialists, doctors, nurses, and allied health professionals, and aims to produce a coordinated and integrated care plan for treatment, rehabilitation, support, and long-term care with active engagement of the older adults in decision-making.

Over the last decade, CGA is being increasingly used in various health conditions and healthcare settings. For example, reviews have assessed the effectiveness of CGA across various care settings including emergency departments ^7– 9^, primary/outpatient care ^10, 11^, community settings ^5, 12^, hospital inpatient departments ^13, 14^, acute geriatric units ^15, 16^, and long-term care facilities ^17^. Similarly, the effectiveness of CGA in certain population groups, such as surgical patients ^18– 20^, cancer patients ^21, 22^, and in the prevention of delirium ^23^, has been reviewed. The resource implications of CGA can vary between studies due to differences in the settings, target population, or the intensity of the intervention ^24^, resulting in heterogeneity in treatment outcomes ^5^.

Although there is strong evidence to support the clinical effectiveness of CGA in managing older adults living with frailty, health systems are increasingly interested in knowing the situations in which CGA can be implemented most efficiently. To examine efficiency, economic evaluation is defined as a comparative analysis of alternative courses of action in terms of both costs and consequences ^25, 26^. Economic evaluation can be classified into two categories: partial and full ^27^. Full economic evaluations constitute cost-utility, cost-effectiveness, cost-consequence, cost-minimization, and cost-benefit analyses. Full economic evaluations compare both the costs and effects of alternative comparisons. The cost of the comparators includes the cost of the intervention (the input cost for comparators) and resource-use consequences (health service utilization after the intervention).


Tc=Ci+Cc

*Where: Tc: Total cost; Ci: Cost of intervention; Cc: resource use consequence*

Effectiveness in the comparison groups can be measured in quality-adjusted life-years (QALYs) or other outcomes such as life-years gained, deaths averted, and improvement in frailty.

With respect to design, economic evaluations can be classified into trial-based and model-based economic evaluations. Trial-based economic evaluation is conducted alongside a randomised controlled trial (RCT) by tracking relevant costs and effectiveness data of comparison groups throughout the trial. Model-based economic evaluations define a set of multiple factors within a single decision-analytic framework, generating expected costs and effects for comparison groups and facilitating decision-making under uncertainty.

As the number of economic evaluations of CGA has increased, systematic reviews have contributed to knowledge by systematically identifying the evidence base and providing critical appraisal of reporting quality ^28, 29^. Previous systematic reviews exist but offer opportunities for improvement:1) Ellis *et al.* ^13^ included economic evaluation of CGA in inpatient settings, but primarily to inform a decision analytic model; 2) Briggs *et al.* ^5^ described the costs of CGA in community settings, but did not critique full economic evaluations; and 3) Garrard *et al.* mentioned the cost-effectiveness of CGA in primary care, but did not critically appraise reporting quality ^10^. To date, we have not found a systematic review or critical appraisal of the full economic evaluation of CGA. Therefore, this protocol aims to appraise full economic evaluation studies on CGA and assess the cost-effectiveness of the intervention by focusing on various care settings, economic perspectives, and sub-group characteristics.

## Methods

The Preferred Reporting Items for Systematic Reviews and Meta-analyses Protocols 2015 (PRISMA-P) was used to develop the protocol ^30^. The PRISMA-P checklist can be found online (see supplementary Appendix 1) ^31^. This review will be conducted and reported in accordance with the 2020 PRISMA guidelines ^32^. This review was registered in PROSPERO (registration number CRD42023492586) on December 11, 2023.

### Criteria for eligibility of studies

***Inclusion criteria***

**Types of studies**: We will include full economic evaluation studies, either model-based or trial-based, conducted in the English language. Studies with at least six months of follow-up (or time horizon) will be included to appropriately capture the impact on costs and effectiveness ^5, 29^.

**Population:** This review will encompass individuals aged 65 years or older (or 55 years or older if the average age of study participants exceeds 70 years) ^5^. The participants may or may not be acutely unwell, and they should be identified as either frail or at risk of adverse outcomes (e.g., falls, functional decline, nursing home admission).

**Intervention:** Comprehensive geriatric assessment provided at all levels of care, including home, nursing home, primary care, and tertiary health facilities by a multidisciplinary team for older adults.

**Comparison:** the comparator is care as usual or standard care

**Outcomes:** The primary outcomes will include the mean costs, mean effects, incremental costs, and incremental effectiveness.

***Setting***

All healthcare settings, including CGA provided at home, GP practice, emergency/acute care departments, outpatient departments, inpatient departments, and long-term care facilities, will be considered.

***Exclusion criteria***

We will exclude studies that do not define population groups as "at risk.” Studies with CGA interventions provided without the explicit involvement of geriatric expertise will not be included. Studies that applied partial economic evaluation will be excluded from the review ^25, 27^. Studies with less than six months of follow-up (time horizon) will be excluded. Commentaries, reviews, methodological articles, and editorials will also be excluded. We will exclude studies that are conducted on a single disease condition ^10^.

Table 1 below presents the inclusion and exclusion criteria.

**Table 1.** Inclusion and exclusion criteria.

Criteria	Inclusion	Exclude
Population	- Older adults ≥65 years (or ≥ 55 years if the average age is >70 years). - Identified as either living with frailty or at risk of adverse outcomes (e.g., falls, functional decline, cognitive decline, nursing home admission	- Population with a specific disease. For example, if CGA is provided to cancer patients. - Population not identified as either living with frailty or at risk of adverse outcomes.
Intervention	- CGA delivered by personnel with training or expertise in gerontology.	- CGA delivered by personnel with no training or expertise in gerontology.
Comparator	- Usual care, standard care, or do-nothing	- Head-to-head comparison. For example, comparing CGA at inpatient versus CGA at home.
Outcome	- Average cost, average effectiveness, AND/OR - Incremental cost and incremental effect AND/OR - Incremental Cost Effectiveness Ratio.	- All outcomes from partial economic evaluations.
Study types	- Full economic evaluations (model-based or trial-based).	- Partial economic evaluation (cost-analysis, cost- description, outcome description). - Commentaries, - Methodological articles
Setting	- All care settings (inpatient, outpatient, GP, Home- based, Emergency, long-term facilities and others).	

### Search methods for identification of studies

Using pilot search terms, a preliminary search was conducted on PROSPERO, PubMed, and Embase to identify any similar systematic reviews, either in progress or published, to mitigate the duplication of work. However, no on-going studies identical to the present systematic review were found.

A search strategy will be devised with support from a librarian at Trinity College Dublin and the literature ^5^. This strategy will be applied across various databases including Embase, MEDLINE, and CINAHL (Supplementary Appendix 2) ^31^. Our approach will include a manual search of reference sections in the identified studies and searches for cited references. Additionally, relevant databases specific to economic evaluations, such as the Tufts CEA Registry and NHS Economic Evaluation Database, will be explored. EndNote 21 will be used to import studies, and Covidence will be utilized to manage search results and remove duplicate records.

### Selection of studies

**Figure 1.**
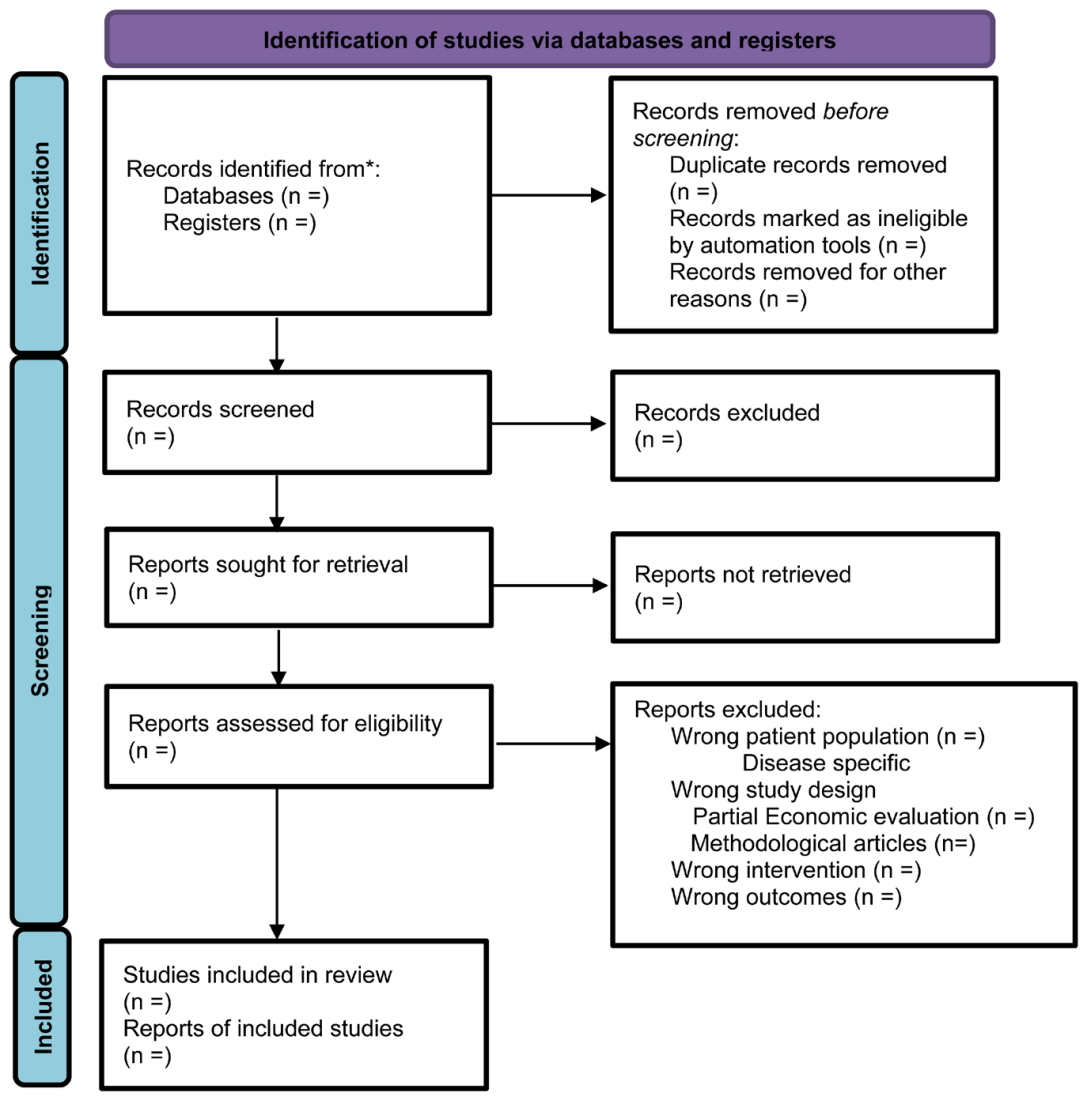
PRISMA flow diagram for the identification of studies.

The study selection will be outlined using a PRISMA flow diagram. Two authors will independently screen the eligible titles and abstracts. The full text of potentially eligible studies will be identified, and a decision to include these full-text articles will be made independently by these two authors. Disagreements to include eligible studies will be approved by discussion in the presence of a third or fourth author (DT or RG). Figure 1 summarises the flow of the selection process.

### Data extraction and management

Two reviewers independently will extract data from selected studies using a structured data collection form on COVIDENCE after a pilot study. If there are disagreements between the two reviewers, the decision will be made by the third and fourth reviewers.

### Appraisal of quality

The Consolidated Health Economic Evaluation Reporting Standards (CHEERS) statement provides guidelines for proper reporting of economic evaluations ^29^. This statement includes 28 criteria outlining the minimum standards for such reports. The CHEERS-2022 checklist will be used to evaluate the reporting quality of economic evaluations ^33^.

To enhance the interpretability of the reporting quality of papers, we will employ a traffic light color-coding system to visually represent the degree to which the key elements of the CHEERS checklist were addressed:

1. Green: Indicates that all elements were fully addressed.
2. Yellow: Indicates that some parts of the elements were only partially addressed.
3. Red: Indicates that the elements were not addressed at all.
4. Black: Indicates that the elements were not applicable.

**Figure 2.**
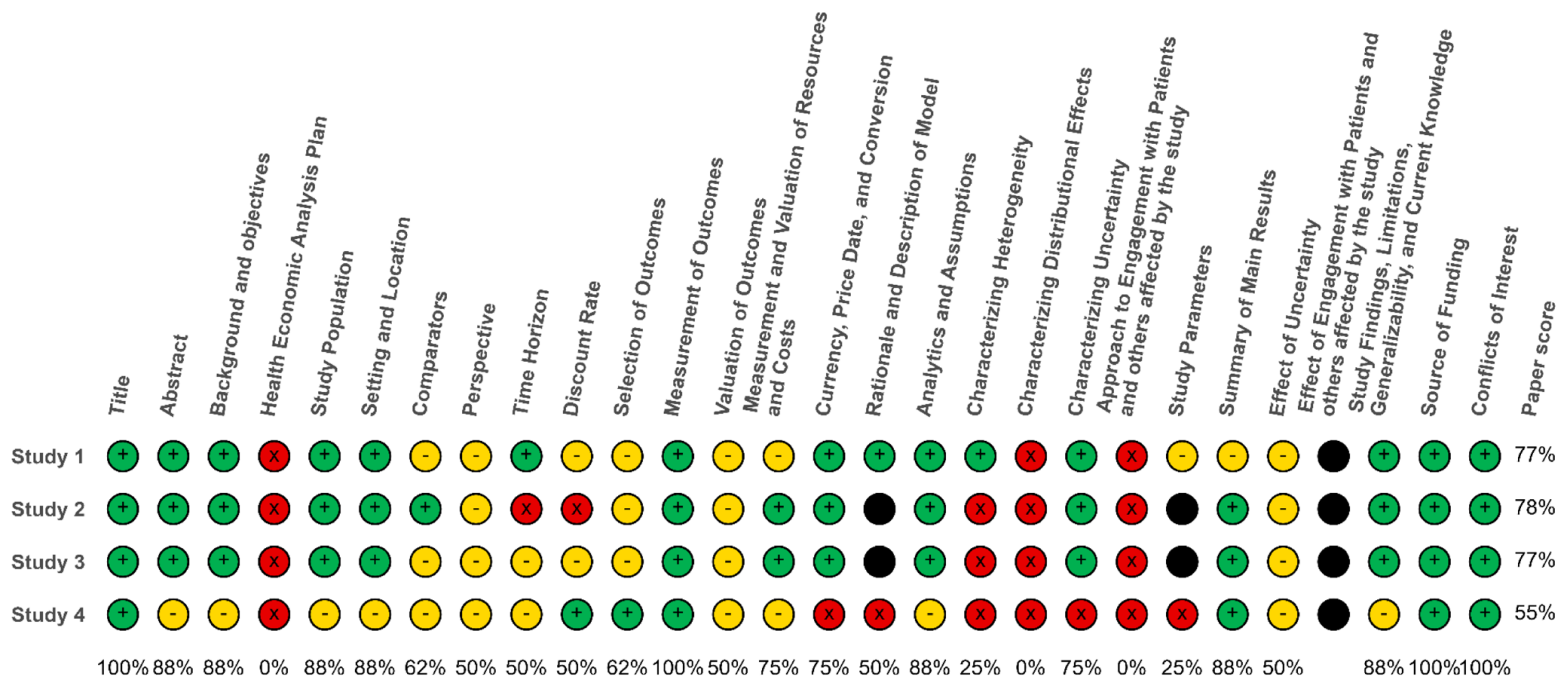
Traffic light system of the reporting quality of the studies.

Figure 2 shows the traffic-light system of the reporting quality of the studies.

### Data analysis and synthesis

A description of the characteristics of the studies and the results will be displayed using a narrative summary and tables. The general characteristics of the included studies will be presented in Table 2.

**Table 2.** General characteristics of the studies.

Characteristics	Description of characteristics	Number	Reference
Year of publication			
Country of study			
Setting			
Target population			
Study design			
Type of economic evaluation			
Perspective of the study			
Time horizon			
Subgroup analyses			
Sponsorship			
Analyses of uncertainty			

**Figure 3.**
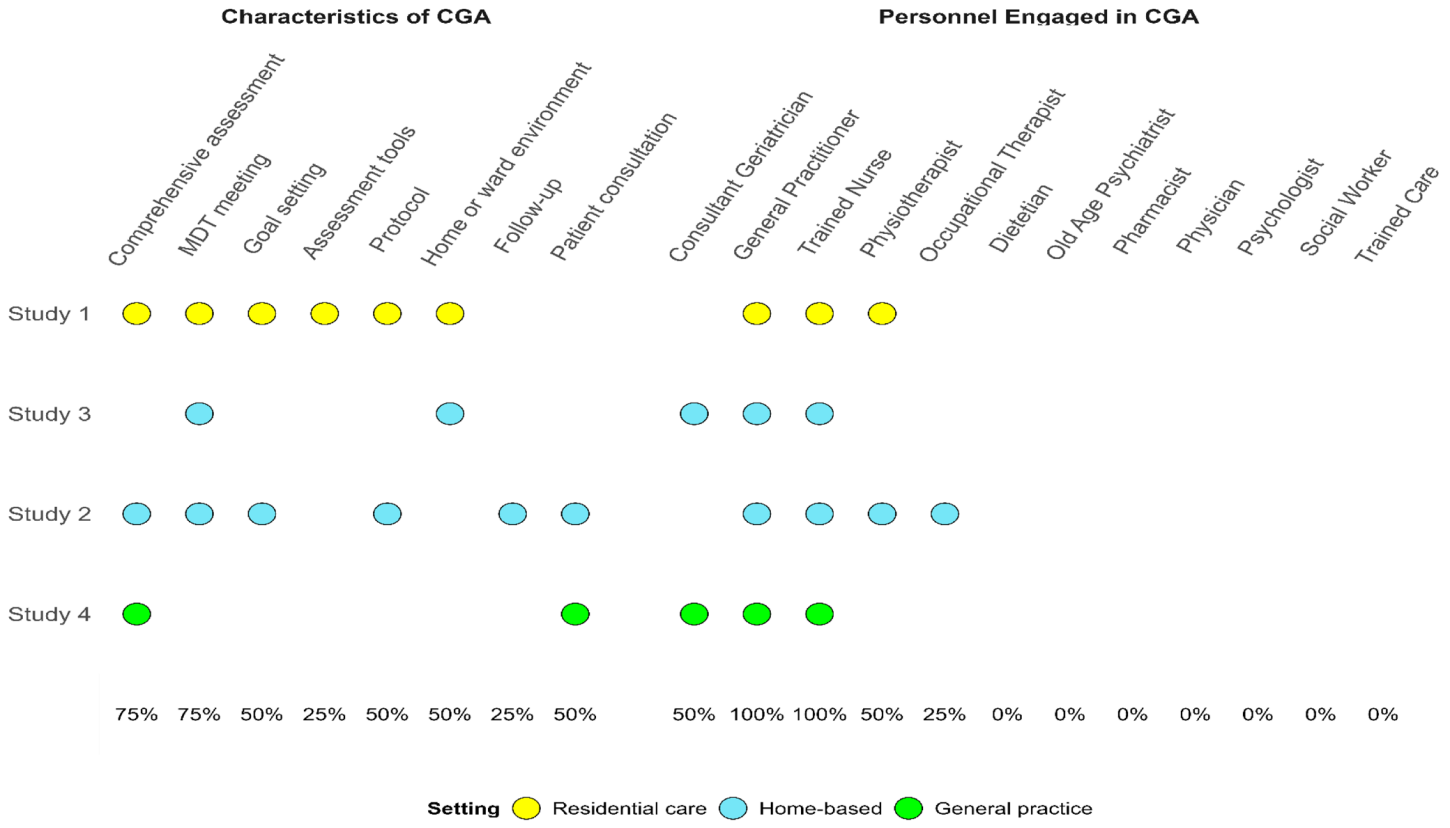
Description of characteristics of interventions.

Narrative reports and tables will be categorized into three sections: intervention characteristics, study characteristics, and study findings. Intervention characteristics encompass the country where the study was conducted, the name of the CGA intervention being investigated, specifics about the services provided to the CGA group, the composition of the team delivering the CGA intervention, details about the target population, and care settings (such as outpatient, inpatient, emergency, and home long-term care facilities). Figure 3 shows a description of the characteristics of the interventions being compared.

Study characteristics include the analytical approach (trial-based or model-based), the health economic perspective of the study, the resources used to measure costs, the time horizon, discount rates for both cost and effectiveness, outcomes measured, the instruments used to measure these outcomes, how outcomes are valued, the threshold applied, and the analysis of uncertainty.

**Figure 4.**
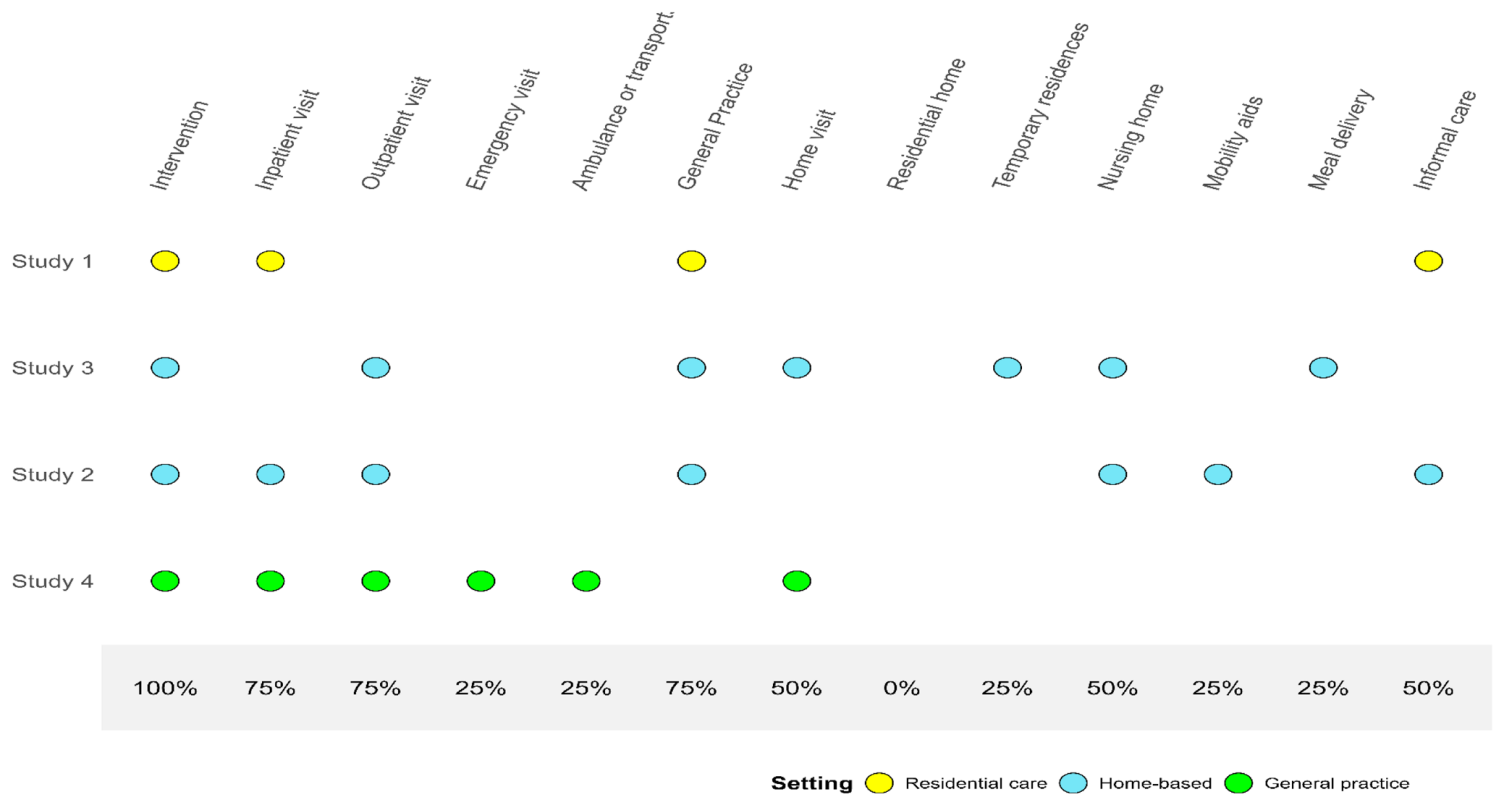
Measurements of health service utilisation for comparison groups.

Descriptions of resource use (health service utilization) for both groups are presented in Figure 4.

A description of the presentation of study characteristics is shown in Table 3.

**Table 3.** Description of characteristics of the studies.

Study ID	Country of study	Target population	Setting	Analytic approach	Perspective of the study	Time horizon	Outcome	Instrument used to measure outcome	Valuation of outcome
Study 1									
Study 2									
Study 3									
Study 4									
Study 5									

**Figure 5.**
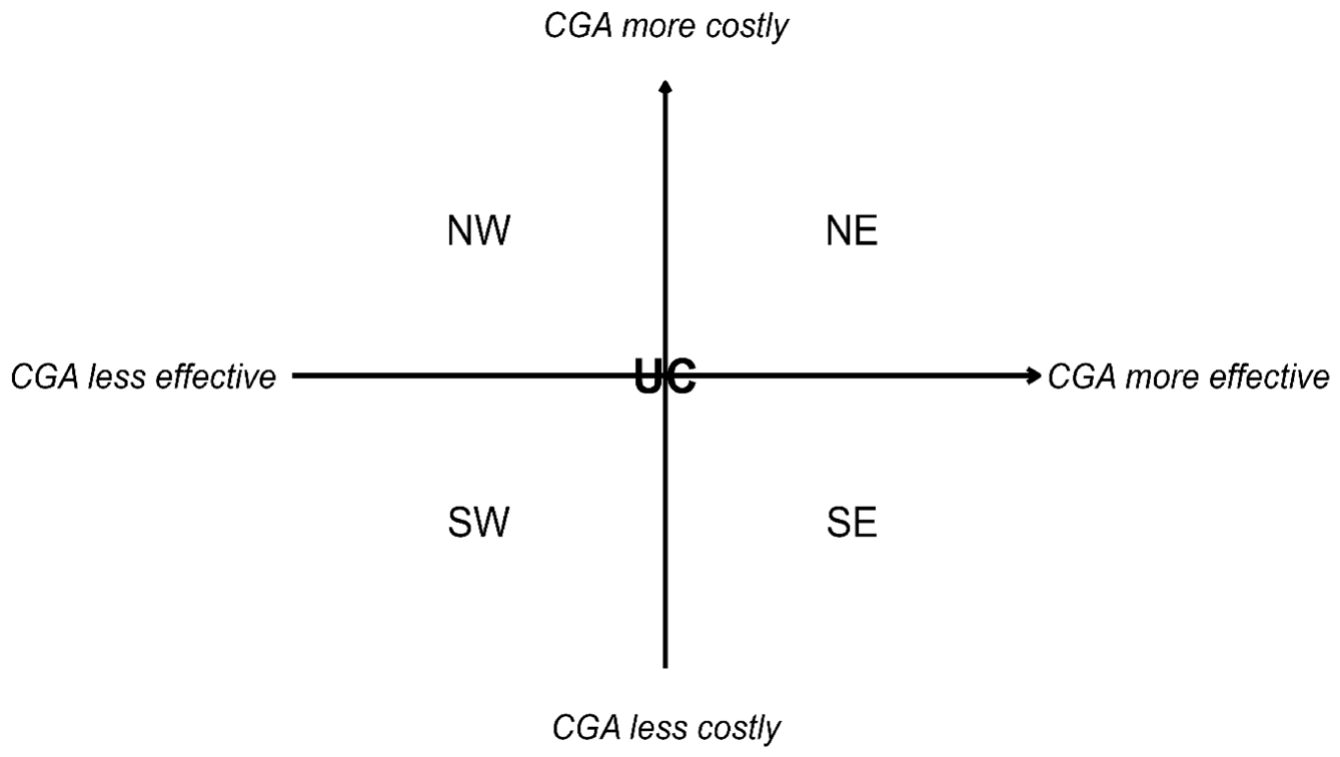
Cost-effectiveness plane for reporting cost-utility studies. NW: Northwest; SW: Southwest; NE: Northeast; SE: Southeast; UC: Usual care.

For cost-utility studies, we will present the difference in costs and quality-adjusted life-years on a cost-effectiveness plane. The cost-effectiveness plane is used to plot the difference in cost and QALY between the intervention and control groups. It is divided into four quadrants: 1) the northeast (NE) quadrant indicates that the intervention is costlier and more effective; 2) the northwest (NW) quadrant indicates higher cost and lower effect; 3) the southwest (SW) quadrant indicates lower cost and lower effect; and 4) the SE quadrant indicates lower cost and lower effect. To allow comparability, all costs reported in the cost-effectiveness plane will be converted to and the price year of 2024 in Euro using the purchasing power parity and gross domestic product deflator index ^34^. Figure 5 illustrates the cost-effectiveness plane that will be used to report the incremental costs and QALYs by care setting.

A detailed description of the cost-utility analysis, including the country of study, setting, base-year currency, willingness to pay per QALY, incremental QALY (95% CI), perspective, incremental cost (95% CI), and the authors’ conclusion about the intervention, will be presented in Table 4.

**Table 4.** Description of results of from the studies.

Author, country	Setting	Base year, currency	WTP per QALY	Incremental QALY (95%CI)	Perspective	Incremental cost (95%CI)	Authors’ conclusion about the intervention
Study 1							
Study 2							
Study 3							
Study 4							

WTP: willingness to pay

### Patient and public involvement

Patients and the public will not participate in the design, execution, reporting, or dissemination of this research.

## Discussion

This review protocol aims to evaluate the cost-effectiveness of CGA. This systematic review will support decision making by assessing and appraising cost-effectiveness across different characteristics, including different care settings and economic perspectives. Reporting summary findings with attention to intervention characteristics, study characteristics, and results will facilitate appraisal and a robust interpretation of the findings. We will appraise approaches to the identification, measurement, and valuation of costs and outcomes which will support proper interpretation of the cost-effectiveness evidence and guide the conduct of future economic evaluations ^35^.

## Ethics and dissemination plan

Ethics approval is not required for this systematic review, as it solely involves the use of publicly available economic evaluation studies and does not involve individual patient data. Therefore, there are no ethical concerns regarding patient confidentiality or informed consent. The findings of this review will be disseminated through presentations at national and international conferences, to reach a wide audience of researchers and practitioners. Additionally, the results will be published in a peer-reviewed journal to contribute to academic literature and inform future research and policymaking in the field of economic evaluation.

## Abbreviations

CHEERS, Consolidated Health Economic Evaluation Reporting Standards; CGA, Comprehensive Geriatric Assessment; CPI, Consumer Price Index; DALY, Disability Adjusted Life Year; ICER, incremental cost-effectiveness ratio; QALY, quality-adjusted life year.

## Data Availability

No data are associated with this article. Figshare: Evaluating the Cost-Effectiveness of Comprehensive Geriatric Assessment: Protocol for a Systematic Review of Economic Evaluations, https://doi.org/10.6084/m9.figshare.28239149 ^36^ The project contains the following extended data Supplementary Appendix 2-Search strategy Data are available under the terms of the Creative Commons Attribution 4.0 International license (CC-BY 4.0). Figshare: PRISMA -P checklist for ‘Evaluating the Cost-Effectiveness of Comprehensive Geriatric Assessment: Protocol for a Systematic Review of Economic Evaluations’, https://doi.org/10.6084/m9.figshare.28239149 ^36^ Data are available under the terms of the Creative Commons Attribution 4.0 International license (CC-BY 4.0).
