# Supplementary material for: Evaluating the Cost-Effectiveness of Comprehensive Geriatric Assessment: Protocol for a Systematic Review of Economic Evaluations
Source: HRB Open Res. 2025 Nov 11;8:26. Originally published 2025 Feb 5. [Version 2] doi: 10.12688/hrbopenres.14066.2 (PMC12982992; doi:10.12688/hrbopenres.14066.2)
Supplement: Peer review history

# Reviewer report: Reviewer response for version 2

**DOI:** 10.21956/hrbopenres.15710.r53783

**Published:** 2026-03-12

**Authors:** Clarabelle T Pham

**Affiliations:** 1 Flinders University, Bedford Park, Australia

Thank you for the opportunity to review this protocol. The protocol is clear and well described. I have 3 comments regarding the methods.

 1. **Methods > Exclusion criteria**: I am unclear why you need to state the exclusion of studies that do not define population groups as "at risk". My understanding is that an individual had to be at risk of frailty or other adverse outcomes to be eligible for CGA. Unless there are countries where they routinely undertake CGA in older adults. 

 2. **Methods > Exclusion criteria**: The exclusion of studies with less than 6 months of follow-up is still in this paragraph but the amendment statement describes that you have no longer excluded based on this criterion. 

 3. **Methods > Appraisal of quality**: Please provide justification for assessing the quality of reporting instead of the quality of methods.

 The CHEERS checklist is guidance for the reporting of EE but it is not a tool to assess the quality and methodological rigour of an EE, such as the CHEC checklist or Drummond's checklist.

 For example, CHEERS asks whether an item was reported but CHEC will ask whether the item reported is appropriate. Using just the CHEERS checklist will only allow you to make conclusions regarding how well studies have reported EE but you will not be able to make any assessments regarding the "appraisal of approaches to the identification, measurement,

 and valuation of costs and outcomes which will support proper interpretation" that you state in your discussion.

Is the study design appropriate for the research question?

Partly

Is the rationale for, and objectives of, the study clearly described?

Yes

Are sufficient details of the methods provided to allow replication by others?

Yes

Are the datasets clearly presented in a useable and accessible format?

Yes

Reviewer Expertise:

I am a health economist with expertise in systematic reviews and economic evaluation methods.

I confirm that I have read this submission and believe that I have an appropriate level of expertise to confirm that it is of an acceptable scientific standard, however I have significant reservations, as outlined above.

# Reviewer report: Reviewer response for version 2

**DOI:** 10.21956/hrbopenres.15710.r51374

**Published:** 2025-11-25

**Authors:** Jonas Ammundsen Ipsen

**Affiliations:** 1 Lillebaelt Hospital, University Hospital of Southern Denmark,, Kolding, Denmark

I have read the comments and changes and the I believe the authors have clarified my remarks sufficiently.

Is the study design appropriate for the research question?

Yes

Is the rationale for, and objectives of, the study clearly described?

Yes

Are sufficient details of the methods provided to allow replication by others?

Partly

Are the datasets clearly presented in a useable and accessible format?

Not applicable

Reviewer Expertise:

NA

I confirm that I have read this submission and believe that I have an appropriate level of expertise to confirm that it is of an acceptable scientific standard.

# Reviewer report: Reviewer response for version 1

**DOI:** 10.21956/hrbopenres.15448.r45708

**Published:** 2025-03-12

**Authors:** Robert Murphy

**Affiliations:** 1 Galway University Hospital, Galway, Ireland

Thank you for the opportunity to review this paper. I am not an expert in economic evaluations but have given my perspective on this from a Geriatric Physician viewpoint and I do think this will be a valuable addition to the literature when considering the cost effectiveness of CGA in different settings. I have a few small suggestions. 

 Abstract: 

 I think the last sentence in the background of the abstract could mention that economic evaluations are being specifically looked at. In the first section of the methods and analysis in the abstract the average reader who is not familiar with economic evaluations might not understand the difference between full and partial. 

 Main Text: 

 Background: 

 Suggest rewording the end of the second sentence for better clarity: "research is increasingly focused on identifying to improve that improve healthy ageing".

 I don't think "frailty" in the second paragraph needs to be in "".

 The wording of the last sentence in paragraph three could be improved. I suggest to simplify it to make the point that costs can differ from study to study because of the different settings CGA is used in. 

 The last section of the last sentence in the background could be a bit clearer from a wording point of view - it is not immediately clear to me what 'explain plans to seek optimal configuration of CGA means' 

 I think in the background you could include an explanation of what is the difference between model based or trial based economic evaluations. 

 Methods: 

 I think you could include in the methods section that you have it registered on PROSPERO and include what date you registered on PROPERO. 

 Population: For the frailty inclusion criteria does there need to be a standardised tool to measure frailty? I wonder about also including at risk of cognitive decline as one of the adverse outcomes. 

 Outcome: Is there one main primary outcome? Or is the primary outcome a composite of the "mean costs, mean effects, incremental costs, and incremental effectiveness". 

 Exclusion Criteria; could you explain a bit more what is meant by Geriatric expertise - is this that the explicit involvement of a Geriatric medicine physician? Or is it the members of the MDT must have specific geriatric medicine training? I know that some (not all) models of CGA do not include a geriatric medicine physician but the rest of a team might have extensive geriatric training which is why I suggest you make this a little clearer. 

 In the search methods for identification of studies section there is some interspersing of tenses: some in the past tense and some in the future tense which makes it read a little disjointed. This is also the case in some other sections of the methods e.g. in the appraisal of quality.

Is the study design appropriate for the research question?

Yes

Is the rationale for, and objectives of, the study clearly described?

Yes

Are sufficient details of the methods provided to allow replication by others?

Yes

Are the datasets clearly presented in a useable and accessible format?

Yes

Reviewer Expertise:

Geriatric Medidine

I confirm that I have read this submission and believe that I have an appropriate level of expertise to confirm that it is of an acceptable scientific standard, however I have significant reservations, as outlined above.

# Response

**Published:** 2025-10-21

**Authors:** Amanuel Yigezu

**Affiliations:** Psychiatry, Trinity College Dublin School of Medicine, Dublin, Dublin 2, Ireland

Dear Reviewer,

 We sincerely appreciate your thoughtful and constructive feedback, which has significantly enhanced the quality of our manuscript. In response, we have carefully considered each comment and revised the protocol to improve its clarity, methodological transparency, and adherence to reporting standards. Presented below is our detailed point-by-point response to your comments.

**Abstract**

- *I think the last sentence in the background of the abstract could mention that economic evaluations are being specifically looked at.*

**Authors’ response: **We thank the reviewer for the comments. We have revised the section as: "This systematic review aims to identify, appraise, and synthesize economic evaluation studies on CGA across various care settings. *”*

- *In the first section of the methods and analysis in the abstract the average reader who is not familiar with economic evaluations might not understand the difference between full and partial.*

**Authors’ response: **Thank you. We have revised the section as:

 Full economic evaluation studies (comparing both the costs and the consequences of alternatives) on CGA will be searched for in the Embase, Medline, CINAHL, CEA registry, and NHSEED databases.

**Main text **

- *Suggest rewording the end of the second sentence for better clarity: "research is increasingly focused on identifying to improve that improve healthy ageing".*

**Authors’ response: **Thank you. We have revised the sentence as:

 With increasing life expectancy and changes in demography (1), the demand for healthcare is increasingly rapidly in line with the growth of the older population (2). The World Health Organization (WHO) defines "healthy ageing" as "the process of developing and maintaining the functional ability that enables well-being in older age" (3)”.To realize healthy aging, health and social care systems advocate a shift from acute management of cases to coordinated, longitudinal, and integrated care for older adults (4).  As a result, researchers are increasingly focused on identifying interventions that improve healthy ageing.

- *I don't think "frailty" in the second paragraph needs to be in "".*

**Authors’ response: **Thank you. We have revised the sentence as:

 CGA is considered for older adults living with frailty or at risk of adverse outcomes.

- *The wording of the last sentence in paragraph three could be improved. I suggest to simplify it to make the point that costs can differ from study to study because of the different settings CGA is used in.*

**Authors’ response:** *Thank you. We have revised the sentence as follows:*

 The resource implications of CGA can vary between studies due to differences in the settings, the target population, or the intensity of the intervention, resulting in heterogeneity in treatment outcomes.

- *The last section of the last sentence in the background could be a bit clearer from a wording point of view - it is not immediately clear to me what 'explain plans to seek optimal configuration of CGA means'.*

**Authors’ response: **Thank you. We have revised the sentence as follows:

 Therefore, this protocol aims to appraise full economic evaluation studies on CGA and assess the cost-effectiveness of the intervention by focusing on various care settings, economic perspectives, and sub-group characteristics. 

- *I think in the background you could include an explanation of what is the difference between model based or trial based economic evaluations.*

**Authors’ response:** *Thank you. We have included about model-based and trial based economic evaluations. *With respect to design, economic evaluations can be classified into trial-based and model-based economic evaluations. Trial-based economic evaluation is conducted alongside a randomised controlled trial (RCT) by tracking relevant costs and effectiveness data of comparison groups throughout the trial. Model-based economic evaluations define a set of multiple factors within a single decision-analytic framework, generating expected costs and effects for comparison groups and facilitating decision-making under uncertainty

- *I think you could include in the methods section that you have it registered on PROSPERO and include what date you registered on PROPERO.*

**Authors’ response:** **Thank you. We have added the Prospero registration detail.**

 This review was registered in PROSPERO (registration number CRD42023492586) on December 11, 2023.

- *Population: For the frailty inclusion criteria does there need to be a standardised tool to measure frailty? I wonder about also including at risk of cognitive decline as one of the adverse outcomes.*

**Authors’ response: **Thank you, we have included cognitive decline as an example of population at risk of adverse outcome.

- *Outcome: Is there one main primary outcome? Or is the primary outcome a composite of the "mean costs, mean effects, incremental costs, and incremental effectiveness". *

**Authors’ response: **Thank you. We have used AND/OR to show it is either or combination of the lists.

- *Exclusion Criteria; could you explain a bit more what is meant by Geriatric expertise - is this that the explicit involvement of a Geriatric medicine physician? Or is it the members of the MDT must have specific geriatric medicine training? I know that some (not all) models of CGA do not include a geriatric medicine physician but the rest of a team might have extensive geriatric training which is why I suggest you make this a little clearer. *

**Authors’ response:** *Thank you. We have revised the criteria as “*CGA delivered by personnel with no training or expertise in gerontology *”.*

- *In the search methods for identification of studies section there is some interspersing of tenses: some in the past tense and some in the future tense which makes it read a little disjointed. This is also the case in some other sections of the methods e.g. in the appraisal of quality.*

**Authors’ response: **Thank you. We have revised the two sentences to:

 “A search strategy will be devised with support from a librarian at Trinity College Dublin and the literature. This strategy will be applied across various databases including Embase, MEDLINE, and CINAHL (Supplementary Appendix 2). Our approach will include a manual search of reference sections in the identified studies and searches for cited references. Additionally, relevant databases specific to economic evaluations, such as the Tufts CEA Registry and NHS Economic Evaluation Database, will be explored. EndNote 21 will be used to import studies, and Covidence will be utilized to manage search results and remove duplicate records.”

 “To enhance the interpretability of the reporting quality of papers, we will employ a traffic light color-coding system to visually represent the degree to which the key elements of the CHEERS checklist were addressed:”

# Reviewer report: Reviewer response for version 1

**DOI:** 10.21956/hrbopenres.15448.r45710

**Published:** 2025-02-14

**Authors:** Jonas Ammundsen Ipsen

**Affiliations:** 1 Lillebaelt Hospital, University Hospital of Southern Denmark,, Kolding, Denmark

Dear Authors

 Thank you for giving me the opportunity to review your protocol addressing the interesting subject of cost-effectiveness of comprehensive geriatric care assessment across varius health care setting. it is an relevant research question and I look forward to reading the review when completed.

  The protocol is well-written, transparent, and easy to follow. I do, however, have some comments and suggestions for your consideration.

1. Is the rationale for, and objectives of, the study clearly described?  
  1. I believe the rationale and objectives are well argumented. I only have a minor comment regarding your aim. I believe this sentence are a bit broad: “and explain plans to seek optimal configurations of CGA” I would suggest you clearly state the aim of the review instead.
2. Is the study design appropriate for the research question?
  1. Study design: the study design are appropriate, however I recommend defining how the synthesis of economic evaluations will be completed.    
  2. Inclusion criteria: I acknowledge that Briggs et al also used the six month criteria, but their focus was on community dwelling high risk patients. I understood from you aim that you focussed on a broader type of setting. Hence, you may need a bit wider inclusion criteria to reduce the risk of missing potentially relevant studies.
  3. Population: I not sure what the argument are for potentially including 55 years old are. CGA are an intervention intended for the geriatric patients. so I would expect the mean age to reflect that.
  4. Intervention: I believe the protocol would benefit from a more in-depth description of the intervention and in which settings it has found use.
  5. Outcomes: I suggest defining one primary outcome for instance the incremental cost-effectiveness ratio and the remaining as secondary outcomes.     
  6. Setting: There are large differences in the health care setting around the world, providing some description or definition on the different healthcare settings may be helpful for readers: CGA provided at home, GP practice, emergency/acute care departments, outpatient departments, inpatient departments, and long-term care facilities.
  7. This may be a discussion topic or something that’s needs to be addressed in the protocol. But how will you group and compare countries across health care systems. In Scandinavia and the commonwealth there are an publicly financed single payer system while the US operates with an insurance based system. This will affect costs.  
  8. Appraisal of Quality: The CHEERS guideline is designed to ensure reporting, however to my knowledge it is not intended for Risk of Bias assessment. Meaby you could look into the: Quality of Health Economic Studies (QHES) Instrument, Drummond checklists or another that is designed for quality assessment of economic evaluations. Philips et al. Model Assessment Criteria is designed for model based economic evaluation. Watts et al. published an article on the subject in 2019 that may be worth looking in to Use of Checklists in Reviews of Health Economic Evaluations, 2010 to 2018.  
  9. You may encounter publications of varying age will you be forward discounting costs?

Is the study design appropriate for the research question?

Yes

Is the rationale for, and objectives of, the study clearly described?

Yes

Are sufficient details of the methods provided to allow replication by others?

Partly

Are the datasets clearly presented in a useable and accessible format?

Not applicable

Reviewer Expertise:

Cost-effectiveness, hip fracture, rehabilitiation

I confirm that I have read this submission and believe that I have an appropriate level of expertise to confirm that it is of an acceptable scientific standard, however I have significant reservations, as outlined above.

# Response

**Published:** 2025-10-21

**Authors:** Amanuel Yigezu

**Affiliations:** Psychiatry, Trinity College Dublin School of Medicine, Dublin, Dublin 2, Ireland

Dear reviewer,

 We sincerely thank the reviewers for their thoughtful and constructive feedback, which has greatly strengthened our manuscript. We have carefully addressed all comments and revised the protocol to improve clarity, methodological transparency, and alignment with reporting standards. Below is our detailed point-by-point response to the comments.

 Title **"** **Evaluating the Cost-Effectiveness of Comprehensive Geriatric Assessment: Protocol for a Systematic Review of Economic Evaluations.**

*1. I believe the rationale and objectives are well argumented. I only have a minor comment regarding your aim. I believe this sentence are a bit broad: “and explain plans to seek optimal configurations of CGA” I would suggest you clearly state the aim of the review instead.* **Authors’ response: **Thank you. We have revised the sentence as follows:

 Therefore, this protocol aims to appraise full economic evaluation studies on CGA and assess the cost-effectiveness of the intervention by focusing on various care settings, economic perspectives, and sub-group characteristics.

 2. *Study design: the study design are appropriate, however I recommend defining how the synthesis of economic evaluations will be completed.*

**Authors’ response** *:Thank you. We plan to change the price year of the costs to 2024 to compare cost-utility studies on cost-effectiveness plane. *To allow comparability, all costs reported in the cost-effectiveness plane will be converted to and the price year of 2024 in Euro using the purchasing power parity and gross domestic product deflator index

*3. Inclusion criteria: I acknowledge that Briggs et al also used the six month criteria, but their focus was on community dwelling high risk patients. I understood from you aim that you focussed on a broader type of setting. Hence, you may need a bit wider inclusion criteria to reduce the risk of missing potentially relevant studies.​​​*

*​​​​* **Authors’ response: **Thank you for your recommendations

 We have now revised the exclusion criteria and included the studies with time horizon less than 6 months.

*4. Population: I not sure what the argument are for potentially including 55 years old are. CGA are an intervention intended for the geriatric patients. so I would expect the mean age to reflect that.*

**Authors’ response:** **Thank you.**

 We acknowledge the comments of the reviewer. However, we have used similar inclusion criteria used by the Cochrane review by Briggs et al.

*5. Intervention: I believe the protocol would benefit from a more in-depth description of the intervention and in which settings it has found use.*

**Authors’ response: **We thank the author for the comment. We have tried to revise the description of CGA by stating, for which population it is indicated, its characteristics including multidimensional assessment, interdisciplinary team, and care planning with active engagement of the older adults.

 “Comprehensive Geriatric Assessment (CGA) is a multidimensional interdisciplinary diagnostic process focused on determining an older person's medical, psychological, and functional capabilities to develop a coordinated and integrated care plan (5). CGA is often deployed considered for older adults living with frailty or at risk of adverse outcomes. when older people are identified as at risk of "frailty" (6). To improve the trajectory of aging, CGA considers various factors, including medical, psychological, and functional impairment, as well as environmental and social issues. CGA requires an interdisciplinary team that may include geriatric specialists, doctors, nurses, and allied health professionals, and aims to produce a coordinated and integrated care plan for treatment, rehabilitation, support, and long-term care with active engagement of the older adults in decision-making.”

 We acknowledge the comments and believe that we have already addressed reviews on CGA that consider various settings and patient populations. However, we have not provided a detailed account of where CGA has been found useful, as it is beyond the scope of this review.

*6. Outcomes: I suggest defining one primary outcome for instance the incremental cost-effectiveness ratio and the remaining as secondary outcomes.*

**Authors’ response:** *We thank the reviewer for the comment.*

*We have now improved it to reporting at least one of*

*Average cost, average effectiveness,*

*AND/OR*

*Incremental cost and incremental effect*

*AND/OR*

*Incremental Cost Effectiveness Ratio.*

*7. Setting: There are large differences in the health care setting around the world, providing some description or definition on the different healthcare settings may be helpful for readers: CGA provided at home, GP practice, emergency/acute care departments, outpatient departments, inpatient departments, and long-term care facilities.*

**Authors’ response:** *We thank the reviewer for this helpful comment. Given the considerable variation in healthcare settings across countries, it may be challenging to provide a comprehensive global description. However, we plan to include detailed descriptions of how the intervention is delivered in each study, which will help clarify the specific settings in which CGA is implemented.*

*8. This may be a discussion topic or something that’s needs to be addressed in the protocol. But how will you group and compare countries across health care systems. In Scandinavia and the commonwealth there are an publicly financed single payer system while the US operates with an insurance based system. This will affect costs.*

**Authors’ response: **We thank the reviewer for this important observation. We acknowledge that healthcare system differences can significantly influence costs. To improve the interpretation of our findings, we plan to include the country in which each study was conducted in the review.

*9. Appraisal of Quality: The CHEERS guideline is designed to ensure reporting, however to my knowledge it is not intended for Risk of Bias assessment. Meaby you could look into the: Quality of Health Economic Studies (QHES) Instrument, Drummond checklists or another that is designed for quality assessment of economic evaluations. Philips et al. Model Assessment Criteria is designed for model based economic evaluation. Watts et al. published an article on the subject in 2019 that may be worth looking in to* Use of Checklists in Reviews of Health Economic Evaluations, 2010 to 2018.  

**Authors’ response: **We thank the reviewer for this valuable comment. We acknowledge that the CHEERS checklist is intended to assess the reporting quality of economic evaluations rather than risk of bias. At this stage, we do not plan to include a separate methodological quality assessment for the economic evaluations themselves and will use CHEERS solely to evaluate reporting quality. However, we have removed the ROB-1 assessment from our analysis.  We will also acknowledge this limitation in the final review.

*10. You may encounter publications of varying age will you be forward discounting costs?* **Authors’ response: **We thank the reviewer for this comment. In our synthesis, we plan to adjust all reported costs to a common price year to facilitate comparison, particularly for visualizing cost-utility analyses on the cost-effectiveness plane. The remainder of the review will primarily involve a descriptive and narrative synthesis.
